# Lord Lister

**Published:** 1912-03

**Authors:** 


Lord Lister, was born April 5, 1827. He took his degree in arts at the University of London, in 1847 and his medical degree in 1854. He attained eminence as a surgeon in Edinburgh and later in London, and was surgeon to Queen Victoria. He was made a baronet in 1883 and was raised to the peerage as the first Baron Lister, in 1897. He was born of Quaker parents, received his early training at the School of Friends, and was throughout his life, a member of the Society of Friends. He died without heirs, Feb. 11, 1912, aged nearly 85. While it cannot be claimed that he either discovered bacteria nor recognized the relation of certain bacteria to pathologic processes, nor even that he was alone in the early recognition, of the necessity of cleanliness and of chemic assistance in preventing sepsis, it may fairly be claimed that modern surgery owes to him the conquest of sepsis and, hence, its possibility of development in directions and to a length otherwise impossible. His comprehensive grasp of the practical importance of pyogenic germs, his reduction of the problem to a definite means of solution, his elaboration of a practical system, his general surgical skill, scientific and literary ability, and later, his well earned prestige, enabled him to bring about convincing results. We do not overlook the fact that in details, his methods were, especially in the beginning, cumbersome and somewhat dangerous. For years "Listerism" was the subject of attack by those who had simplified antiseptic methods while a long battle has been waged between adherents of antisepsis and of asepsis which is now just about at its conclusion, as the result of accumulated experience, some of it dearly bought, as to the practical lines to be drawn between negative and positive, thermic and chemic, extrinsic and vital means of securing the same ultimate object.

But, now that a general agreement has been reached as to the best practical means of nullifying pyogenic processes, we must recognize that these points of difference were merely minor conflicts in the same camp and that success in the entire campaign against surgical infections has been due essentially to the general principles taught by Lord Lister. Even if his arguments had been addressed to an unprogressive profession, incapable of independent research and thought, even if his system had arisen in a mind itself unprogressive beyond the first point of elaboration of technic, much more than half of the ultimate gain would have been secured. Nor is the credit due Lord Lister eclipsed by the fact that many other workers have attacked the subject from a multitude of aspects, sometimes empirically, and with special reference to puerperal fever, as our own poet-physician, Oliver Wendell Holmes; sometimes with special reference to erysipelas and gangrene; often in recent years from the academic standpoint of bacteriology; often too, with a distinct hostility to the special technic of Lord Lister, and with the special purpose of abrogating the chemic agents introduced by him into a special surgical role.

